# Autophagy as a decisive process for cell death

**DOI:** 10.1038/s12276-020-0455-4

**Published:** 2020-06-26

**Authors:** Seonghee Jung, Hyeonjeong Jeong, Seong-Woon Yu

**Affiliations:** 1 Department of Brain and Cognitive Sciences, Daegu Gyeongbuk Institute of Science and Technology (DGIST), Daegu, 42988 Republic of Korea; 2 Neurometabolomics Research Center, DGIST, Daegu, 42988 Republic of Korea

**Keywords:** Macroautophagy, Macroautophagy

## Abstract

Autophagy is an intracellular catabolic pathway in which cellular constituents are engulfed by autophagosomes and degraded upon autophagosome fusion with lysosomes. Autophagy serves as a major cytoprotective process by maintaining cellular homeostasis and recycling cytoplasmic contents. However, emerging evidence suggests that autophagy is a primary mechanism of cell death (autophagic cell death, ACD) and implicates ACD in several aspects of mammalian physiology, including tumor suppression and psychological disorders. However, little is known about the physiological roles and molecular mechanisms of ACD. In this review, we document examples of ACD and discuss recent progress in our understanding of its molecular mechanisms.

## Cell death: cell component recycling in health and disease

A natural cellular recycling process could be harnessed or targeted for the treatment of multiple diseases including cancer and psychological disorders. Autophagy is a process that occurs within cells, whereby damaged, toxic, or obsolete cellular components are degraded and recycled to release energy and maintain balance. However, scientists now recognize that autophagy can trigger cell death under certain conditions. Seong-Woon Yu and co-workers at the Daegu Gyeongbuk Institute of Science and Technology, South Korea, reviewed recent research into the mechanisms and role of autophagy in health and disease. Autophagic cell death is implicated in the suppression of tumor development; for example, inducing autophagy led to the death of precancerous cells in mice. Autophagy may also regulate immune cell populations, and play a role in the death of brain cells during chronic stress-related disorders.

## Introduction

Autophagy/macroautophagy is a lysosome-dependent catabolic process characterized by increased formation of double-membrane autophagosomes for the sequestration of cytoplasmic components and subsequent degradation after autophagosome fusion with lysosomes^1,2^. Autophagy occurs during normal development at the basal level, as well as under stress conditions. Autophagy is generally considered as a cell survival/protection mechanism because it removes toxic or obsolete proteins and organelles and recycles the degradation products for use as sources for energy and metabolites in anabolic pathways^3^. However, autophagy has also been recognized as a cell death pathway, first in *Drosophila* and recently in mammalian systems^4,5^. Nevertheless, the definition of autophagic cell death (ACD) has been neither universally understood nor unanimously accepted in the field^4^. Therefore, the relationship between autophagy and cell death remains unclear and warrants further study to harness autophagy for the treatment of various human diseases.

Autophagy is induced by adverse environmental conditions, such as starvation, growth factor deprivation, and pathogen infection^6^. Extracellular cues, including those of hormones and cytokines, can also regulate autophagy. For example, Th1 cytokines, including interferon-γ, tumor necrosis factor-α, interleukin (IL)-2, IL-6, and transforming growth factor-β, stimulate autophagy, whereas Th2 cytokines, including IL-4, IL-10, and IL-13, inhibit autophagy and thus regulate inflammatory mediators^7^. Insulin and insulin-like growth factor 1 are known to inhibit autophagy. In a fasting state, increased glucagon and epinephrine and norepinephrine secretion induce autophagy, and glucocorticoids have also been shown to induce autophagy by stimulating the transcription of autophagy genes such as ATG5, LC3, and Beclin-1 in various tissues^8^. Including those of autophagy-inducing signals, the molecular details of autophagy and the techniques to assess autophagy flux have been well documented in other reviews^1,9^. The beneficial roles of autophagy in diverse aspects of human physiology and diseases, including development, metabolism, neurodegeneration, and aging, are also well covered elsewhere^10–13^. In addition, cell death subroutines have been recently classified on the basis of mechanical and molecular aspects of cell death processes^5^. Therefore, in this review, we avoid a lengthy repetition of the description of autophagy and cell death processes and focus on the death-promoting roles of autophagy and the intertwined connection between autophagy and apoptosis. We also present recent findings on the molecular mechanisms underlying ACD.

### Programmed cell death

Programmed cell death (PCD), as described by Lockshin and Williams^14^, is defined as controlled cell death evoked by intracellular systems. PCD has fundamental functions in tissue development and homeostasis, as PCD is activated to sculpt or remove structures, regulate cell numbers, and eliminate unnecessary or dysfunctional cells. Therefore, the abnormal regulation of PCD is associated with numerous human diseases, including cancers and neurodegenerative diseases. The Nomenclature Committee on Cell Death has recently classified 12 major cell death modes^5^. However, the classification of PCD into apoptosis (type I), ACD (type II), and necroptosis (type III)^10,15^ adequately serve for our discussion.

#### Apoptosis

Apoptosis is the most well-known mode of PCD and is characterized by specific morphological and biochemical changes in dying cells, including cell shrinkage, chromatin condensation, nuclear fragmentation, membrane blebbing, and chromosomal DNA cleavage^16,17^. Apoptosis can be categorized into extrinsic and intrinsic pathways^17^. The extrinsic pathway, also known as the death receptor pathway, is stimulated by the binding of death ligands to cognate death receptors, including the tumor necrosis factor receptor and Fas receptor^18^. After ligand binding, a death-inducing signaling complex is formed, and procaspase 8 is activated, followed by the activation of downstream executioner caspases, such as caspases 3 and 7^19^. The intrinsic or mitochondrial pathway is initiated by nonreceptor-mediated cellular stressors such as radiation, hypoxia, DNA damage, and oxidative stress^17^. Cellular stress increases mitochondrial membrane permeability, leading to the release of cytochrome *c* from the mitochondrial intermembrane space into the cytosol. Then, cytochrome *c* binds to apoptotic protease-activating factor-1 (APAF-1) and procaspase 9, forming the apoptosome complex, which activates caspase-9 and then executioner caspases, leading to cell death^20,21^. Extrinsic apoptosis is often interconnected with intrinsic apoptosis through proapoptotic Bcl-2 family members^17^.

#### Necroptosis

Previously, necrosis was regarded as an accidental and uncontrolled form of cell death, but it is now recognized that necrosis can be executed in a controlled manner. Therefore, the term “necroptosis” was coined to reflect its regulated nature^22^. Necroptotic cells show morphological characteristics such as cell swelling and rupture of the plasma membrane, and the presence of necroptotic cells is usually associated with inflammation^23^. Receptor-interacting protein kinases 1 and 3 (RIP1 and RIP3) act as key molecules in necroptosis, and the development of inhibitors specific to these kinases has contributed to the current understanding of the regulated nature of necroptosis^23–25^.

#### ACD

In many cases, dying cells develop autophagosomes, leading to the idea of “autophagic” cell death (ACD). Initially, “ACD” was simply a morphological term to describe dying cells showing features of autophagy without implying a causative role for autophagy in cell death^5^. Autophagy may be activated to overcome cell death; on the other hand, apoptosis may impair autophagy to complete cell death. When autophagic flux is impaired, autophagosome maturation is suspended, and autophagosomes may accumulate^9^. Therefore, the use of “ACD” as a descriptive term without mechanistic implications for the role of autophagy in cell death led to confusion. To make matters more complicated, autophagy may precede and trigger apoptosis or necroptosis, leading to the term “autophagy-mediated cell death”^26^. In autophagy-mediated cell death, autophagy accompanies and is required for the activation of other cell death modes. In these cases, inhibition of autophagy can prevent cell death, even though cell death is not executed through autophagy. Thus, the term “ACD” should be applied only when the following criteria are met: (1) cell death occurs without the involvement of other types of PCD, (2) autophagic flux is elevated, and (3) pharmacological or genetic inhibition of autophagy blocks cell death^27^. In the following section, we minimize the introduction of cases of autophagy-mediated cell death and cell death in which autophagy and apoptosis overlap to focus mainly on authentic examples of ACD.

### ACD in model systems

#### ACD in *Drosophila*

**Fig. 1:**
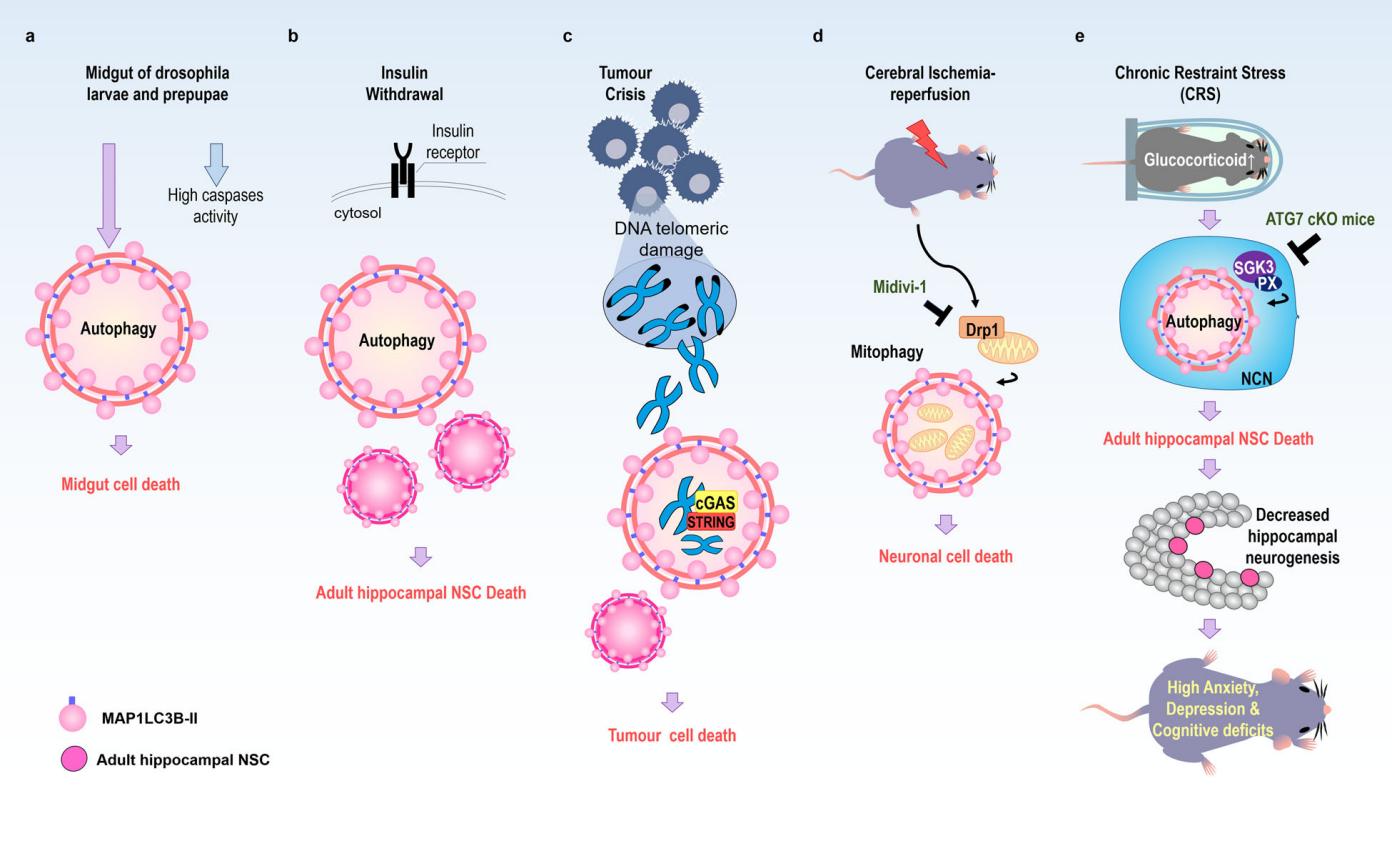
Representative cases of autophagic cell death (ACD). **a** The midgut of *Drosophila* larvae and pupae shows high caspase activity and autophagy flux, but midgut cell death depends only on autophagy. **b** Adult hippocampal neural stem cells (NSCs) undergo ACD following insulin withdrawal without the involvement of apoptosis or necroptosis. **c** During replicative crisis, DNA with telomeric damage is released into the cytosol and is recognized by cGAS and STRING, which induces ACD. **d** Cerebral ischemia–reperfusion induces mitophagy in a DRP1-dependent manner with subsequent neuronal cell death. **e** Chronic restraint stress (CRS) or corticosterone treatment induces ACD in adult hippocampal NSCs via SGK3 in vivo and in vitro. CRS decreases adult hippocampal neurogenesis, which is accompanied by anxiety, depression, and cognitive deficits.

A plausible example of ACD was first presented during developmental cell death in *Drosophila*. In dying larval salivary glands, autophagy-related (*Atg*) genes, as well as apoptotic genes such as those for caspases, are induced, and both autophagy and apoptosis are required for the complete loss of salivary glands^28,29^. Therefore, salivary gland cell death does not meet the criteria of ACD. However, an authentic case of ACD in *Drosophila* was discovered later, in midgut cell death^30^. Decay, a *Drosophila* caspase, is active in the dying midgut and contributes to the activation of other caspases, dronc, and drice1. However, the inhibition of decay or all three caspases cannot block midgut cell death, and the canonical apoptotic pathway is not required for midgut regression. Interestingly, midgut cell death is accompanied by an increase in the number of autophagosomes. Midgut cell death is delayed in *Atg1-* and *Atg18-*mutant flies. Similarly, knocking down *Atg2* and *Atg18* also significantly suppresses cell death in the midgut. Therefore, autophagy, not apoptosis, is crucial for *Drosophila* midgut cell death (Fig. 1a). Midgut removal is a developmental process controlled by the *Drosophila* steroid hormone ecdysone. Decapentaplegic, *Drosophila* bone morphogenic protein/transforming growth factor-β ligand, blocks autophagy-dependent midgut degeneration by inhibiting ecdysone production and thereby impairs the correct timing of development^31^.

#### ACD in *Dictyostelium*

In *Dictyostelium discoideum*, triggering the differentiation of vegetative cells under starvation conditions induces the programmed death of stalk cells, which is characterized by early massive vacuolization and late membrane lesions but intact nuclei and the absence of apoptosis markers such as DNA fragmentation^32^. This developmental cell death requires two successive but separable exogenous signals: (1) starvation/cAMP for the induction of autophagy and (2) differentiation factor DIF-1 for the induction of cell death. Autophagy induced by the first signal does not lead to cell death, and DIF-1 does not induce cell death when added to nonstarved cells. An *Atg1* mutation prevents both starvation-induced autophagy and DIF-1 exposure-induced cell death, showing that autophagy is required for ACD, with Atg1 being a critical inducer^33^. ACD induction by DIF-1 is prevented by mutations in *iplA* (IP3R), *TalB* (talinB), *DcsA* (cellulose synthase), *GbfA, ugpB, glcS* (glycogen synthase), and *atg1*^34^. As mammalian homologs of some of these molecules, such as glycogen synthase kinase 3-beta (GSK3B), also play important roles in ACD (as discussed in the following section), comparative studies between *Dictyostelium* and mammalian cells may provide novel mechanical insights into the molecular and genetic regulation of ACD.

### ACD in mammalian systems

#### ACD in cancer cells

As apoptosis is defective in most cancer cells, due consideration has been given to ACD as an attractive cancer treatment modality to induce PCD by using anticancer agents. ACD has been reported in various cancer cells. In A549 lung cancer cells, resveratrol treatment induced cell death with increased autophagy flux^35^. Cell death occurred in the absence of apoptosis markers, including the cleaved forms of caspases 9, 8, and 3, and *Atg7-*, *Atg12-*, *Beclin-1-*, or *Ulk1*-knockdown increased cell viability and reduced autophagy activation. Signalome-wide screening with shRNAs led to the identification of glucocerebrosidase as a mediator of ACD in resveratrol-treated A549 cells. In pancreatic cancer cells, LZ1, a peptide derived from snake venom cathelicidin, suppressed cell growth both in vitro and in vivo by inducing ACD by binding to and degrading cell surface-expressed nucleolin, subsequently activating AMP-activated protein kinase (AMPK)^36^. As_2_O_3_ specifically triggered ACD in human malignant glioma cells (U373-MG, U251, U87-MG, A172, T98G, and GB1)^37^. An increased number of autophagosomes was apparent after As_2_O_3_ treatment, but no apoptotic features were observed by electron microscopy, and Z-VAD, an apoptosis inhibitor with broad-spectrum caspase inhibition activity, did not prevent As_2_O_3_-induced cell death.

Z-VAD killed mouse L929 fibrosarcoma cells, with the appearance of numerous autophagic vacuoles and morphology that was distinct from that of apoptotic cells^38^. Z-VAD-induced cell death associated with autophagy was also observed in U937 human leukemia cells, a mouse RAW264.7 macrophage cell line, and primary mouse peritoneal macrophages^38^. In this study, pharmacological inhibition and knockdown of *Atg7* and *Beclin-1* inhibited Z-VAD-induced cell death with a parallel decrease in autophagic vacuole formation. These data suggest that autophagy is required for PCD in these various types of cells following Z-VAD treatment. RIP and Jun amino-terminal kinase, but neither p38 nor extracellular signal-regulated kinase, were found to be components of a signaling pathway that led to the activation of autophagy^38^. Of interest, caspase 8 was identified as a target of Z-VAD, as caspase 8 inhibition led to an increase in cell death associated with autophagy features^38^. Further study showed that cell death induced by caspase inhibition is mediated by catalase degradation and subsequent reactive oxygen species (ROS) accumulation, processes that are blocked by autophagy inhibition^39^.

Apoptosis inhibition-induced ACD was also reported in multiple myeloma. In several multiple myeloma cell lines, caspase 10 catalytic activity and cFLIP_L_ expression, driven by IRF4, are required for cell viability irrespective of genetic abnormalities^40^. Caspase 10 inhibition by a broad-spectrum caspase inhibitor, Q-VD-OPH or a more selective caspase 10 inhibitor kills myeloma cells without the hallmarks of apoptosis but through the induction of ACD. Posttranslational cleavage of BCL-2-associated transcription factor 1 (BCLAF1) has been identified in a mechanism of ACD suppression induced by caspase 10. Therefore, overexpression and knockdown of BCLAF1 induced and mitigated ACD, respectively, in multiple myeloma cells with inhibited caspase 10^40^. Apoptosis inhibition-induced ACD may have some clinical relevance when apoptosis is considered as a means of tumor suppression.

In cancer cells, ACD induction by ROS-generating agents has also been observed. In HEK293, U87, and HeLa cells, hydrogen peroxide (H_2_O_2_) and 2-methoxyestradiol (2-ME)-induced ACD, whereas cell death was inhibited by 3-methyladenine (3-MA) or the deletion of *Beclin-1*, *Atg6*, or *Atg7* but not by Z-VAD^41^. Inhibition of the mitochondrial electron transport chain also induced ACD in transformed and cancer cell lines through the generation of ROS^42^. Blocking autophagy failed to reduce ROS generation, positioning ROS upstream of autophagy, which differs from the finding in Z-VAD-induced death of L929 cells^41,42^. Interestingly, neither H_2_O_2_ nor 2-ME could induce autophagy or cell death in mouse primary astrocytes, suggesting a difference in signaling mechanisms between transformed or cancer cells and nontransformed cells^41,42^.

In the HCT116 human colon cancer cell line, oxidative stress leads to acetylation of FoxO1, a forkhead O family protein, by inducing its dissociation from sirtuin-2. Then, acetylated FoxO1 binds to ATG7 in the cytosol, leading to ACD and tumor suppression activity. Regressed tumor growth was observed in a xenograft nude mouse model after transplantation of FoxO1-expressing cancer cells but not after transplantation of FoxO1-expressing cancer cells upon a stable knockdown of *Atg7*, demonstrating that FoxO1 exerts tumor-suppressor activity by inducing ACD^43^.

Neferine, a natural alkaloid isolated from *Nelumbo nucifera*, induces ACD via calcium release after the activation of ryanodine receptor (RYR) and ULK1–PERK and AMPK–mTOR signaling cascades, especially in apoptosis-resistant cancer cell lines, including HeLa, H1299, HepG2, and Lo2 cells^44^. Plasma-activated medium (PAM), which was developed for ovarian cancer suppression, induced ACD in some types of endometrial cancer cells^45^. PAM treatment increased ACD by inactivating the mTOR pathway, providing a potential novel treatment for endometrial cancer.

#### ACD in other mammalian cells

ACD has also been documented in various noncancerous mammalian cell types. Mouse embryonic fibroblasts deficient in both BAX and BAK are resistant to apoptosis and instead undergo nonapoptotic death induced by various apoptotic stimuli^46,47^. This nonapoptotic death is associated with a significant increase in the number of autophagosomes/autolysosomes, can be blocked by the autophagy inhibitor 3-MA or by knockdown of *Atg5* or *Beclin-1*^46,47^, and requires lysosomal membrane permeability^48^.

ACD is the mechanism of PCD in senescent keratinocytes. Keratinocyte senescence is caused by the accumulation of oxidative damage to the nucleus and mitochondria, which can be replicated by applying a subtoxic level of H_2_O_2_^49^. Senescent cells ultimately undergo cell death, not by apoptosis but by ACD^49^.

The number of immune T cells is under tight control through two cell death pathways: (1) activation-induced cell death upon prolonged T-cell receptor activation and (2) activated T-cell-autonomous death. The latter is induced when survival signals, such as those from growth factors, are limited. In activated mouse CD4^+^ T cells, growth factor abrogation-induced cell death can be prevented by blocking autophagy with 3-MA or by knocking down *Beclin-1* or *Atg7*^50^. In addition to the reported involvement of apoptosis and necrosis in the death of activated T cells, this report suggests that autophagy is also important for the regulation of CD4^+^ T-cell homeostasis.

**Fig. 2:**
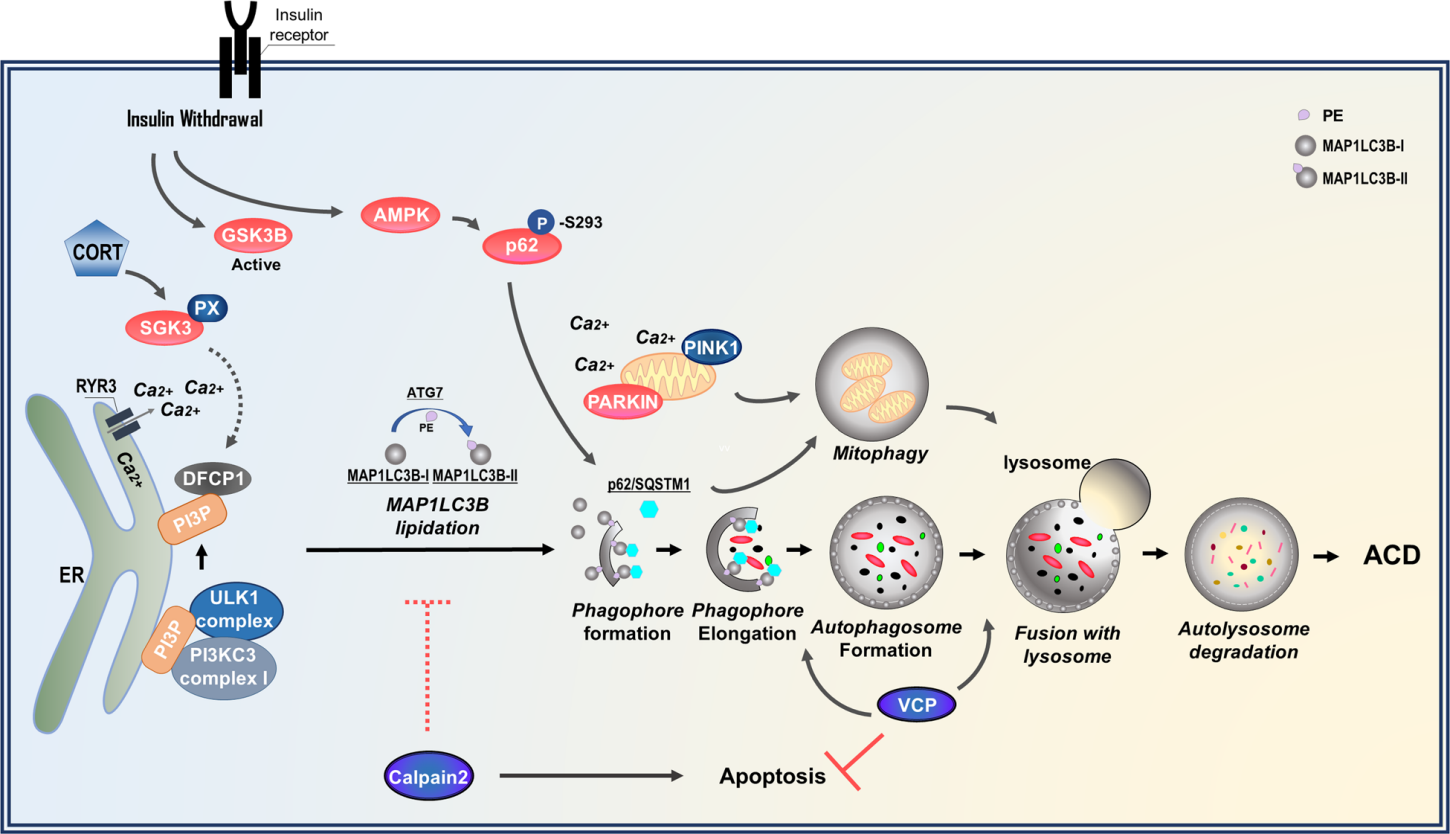
Molecular effectors in ACD of adult hippocampal NSCs following insulin withdrawal or psychological stress. Insulin withdrawal activates GSK3B and AMPK, followed by AMPK-mediated phosphorylation of p62. RYR3 mediates the efflux of Ca^2+^ from the ER. PARKIN levels are upregulated in a GSK3B-dependent manner, and PARKIN is involved in the transfer of ER Ca^2+^ to mitochondria and depolarization of mitochondrial membrane potential. Both p62 and PARKIN promote mitophagy, leading to ACD. Cell death triggered by insulin withdrawal is switched from ACD to apoptosis by calpain 2 and VCP. High levels of corticosterone (CORT) induced by CRS cause ACD via SGK3, which has a PX domain for binding to PI3P and the initiation of autophagy. The dashed lines indicate that the process is not yet experimentally confirmed.

We have also reported ACD in murine adult hippocampal neural stem cells (NSCs) following insulin withdrawal (Fig. 1b)^51^ with no hallmarks of apoptosis (caspase activation, chromosomal DNA fragmentation, or cell death prevention by Z-VAD)^52^. In contrast, insulin-deficient hippocampal NSCs exhibited increased autophagy flux, as determined by assessing morphological and biochemical markers of autophagy. Furthermore, knocking down *Atg7* or promoting autophagy using rapamycin decreased or increased cell death, respectively, fulfilling the criteria for ACD and indicating that this experimental system can be regarded as a genuine model of ACD^27,53^. According to this finding, the molecular machinery of ACD following insulin withdrawal in adult hippocampal NSCs has been gradually established (Fig. 2). GSK3B was identified as one of the upstream kinases involved in the initiation of ACD^52^. Genetic and pharmacological inhibition of GSK3B attenuated ACD, whereas its activation accelerated ACD following insulin withdrawal^52^. This study demonstrated that GSK3B is a positive regulator of ACD following insulin withdrawal in adult hippocampal NSCs. Mitophagy was also observed following insulin withdrawal. Insulin withdrawal activated AMPK, and AMPK phosphorylated p62 at a novel site, Ser-293/Ser-294 (rat/human sequence, respectively)^54^. Phosphorylated p62 was then translocated to mitochondria where it induced mitophagy and ACD^54^. Another important pathway in mitophagy in adult hippocampal NSCs following insulin withdrawal is established by the recruitment of PINK1/PARKIN to depolarized mitochondria^55^. Insulin withdrawal increased the ratio of depolarized mitochondria and their colocalization with autophagosomes. PARKIN was also upregulated in insulin-deprived adult hippocampal NSCs, and it mediated mitophagy and cell death. One interesting role of PARKIN in mitophagy is its mediation of Ca^2+^ transfer from the endoplasmic reticulum (ER) to mitochondria and the induction of mitochondrial depolarization during the early steps of mitophagy. These novel functions of PARKIN, in addition to its well-known role in the recognition and loss of depolarized mitochondria, contributes to mitophagy and cell death in adult hippocampal NSCs^55^. RYR3 is critical for ER calcium release^56^. These studies have firmly established the central role of autophagy in the death of adult hippocampal NSCs upon insulin withdrawal because the effects of GSK3B, RYR3, and PARKIN were all significantly blunted upon *Atg7* knockdown^52–55^.

### ACD in mammalian pathophysiology

#### Tumor suppression

Recently, it was reported that autophagy activation is required for cell death and, thereby, the elimination of precancerous cells during replicative crisis caused by telomere dysfunction and that loss of ACD initiates tumorigenesis in fibroblasts and epithelial cells^57^. During replicative crises, apoptosis markers were not detected, whereas extensive cytoplasmic double-membrane autophagosomes and single-membrane autolysosomes were observed with a reduced level of p62, an autophagy cargo receptor. Moreover, the accumulation of microtubule-associated protein 1 light chain 3-beta-II (MAP1LC3B)-II and p62 after treatment with the autophagy blocker bafilomycin A1 suggested increased autophagy flux during replicative crisis. On the other hand, shRNA against *ATG*3, *ATG*5, or *ATG7* promoted a bypass of the crisis, continued cell proliferation, and increased genome instability. It was also found that telomeric DNA damage activated ACD via the cGAS-STING pathway^57^ (Fig. 1c). These findings highlight autophagy as an essential component in tumor-suppressive mechanisms.

Melanoma cells, which are resistant to apoptosis-inducing drugs, can undergo ACD upon treatment with compounds targeting orphan nuclear receptor TR3^58^. Upon treatment, TR3 is translocated to mitochondria via its interaction with the mitochondrial outer membrane protein Nix and dissipates mitochondrial membrane potential to induce massive mitochondrial clearance and ACD. TR3 translocation-triggered autophagy requires TR3 to cross into the mitochondrial inner membrane; therefore, this nuclear receptor becomes integrated into a mitochondrial signaling pathway to induce ACD. However, further details are not yet clear, particularly those that might indicate whether selective removal of mitochondria through mitophagy is required for ACD or whether mitochondrial clearance is simply part of bulk autophagic degradation. Despite the lack of detailed knowledge of the mode of action of TR3-targeting compounds, engagement of TR3 by compounds targeted to it demonstrated antimelanoma activity in the liver and lung in several mouse models^58^.

The role of autophagy in tumorigenesis is complex, with autophagy having different consequences for cancer development and treatment depending on the types of tumors and their stages^59^. Autophagy may serve as a tumor-suppressor pathway through several mechanisms: by maintaining genomic stability; by eliminating defective subcellular organelles, including depolarized mitochondria, and thus by removing the cellular sources of oxidative stress; and by regulating inflammation. All of these mechanisms may contribute to the prevention of cancer development. However, the survival- and death-promoting functions of autophagy make its association with cancer treatment very complicated. In contrast to the antitumor roles of autophagy, whereby cancer cells are eradicated by ACD, autophagy can maintain cancer cells viability by providing metabolic substrates under nutrient-limited conditions, delaying the onset of apoptosis of cells challenged by chemotherapeutic drugs or irradiation, and enhancing cancer cell survival under stressful microenvironments, including hypoxia^60^.

#### Excitotoxicity

Kainate-induced excitotoxicity combined with hypoxia was used to mimic hypoxia–ischemia in vitro and induced cell death in primary rat cortical neurons. Cell death was blocked by pharmacological autophagy inhibitors and genetic inhibition of autophagy by knocking down *Beclin-1* or *Atg7*, whereas overexpression of Beclin-1 or ATG7 enhanced hypoxic excitotoxicity^61^. In vivo knockdown via intrastriatal injection of lentivirus-expressing sh*Beclin-1* reduced striatal damage in a rat model of neonatal hypoxia–ischemia^61^. No apoptosis activation was observed, and Bcl-2 overexpression or caspase inhibition prevented neuronal cell death.

Peroxynitrite (ONOO^–^), a representative reactive nitrogen species, activates mitophagy via the PINK1/PARKIN pathway to mediate cerebral ischemia–reperfusion injury (Fig. 1d)^62^. Increased nitrotyrosine levels were observed in the plasma of ischemic stroke patients and in ischemia–reperfusion injured rat brains; the recruitment of DRP1 to dysfunctional mitochondria with decreased membrane potential activated the PINK1/PARKIN pathway to initiate mitophagic flux and thus remove the damaged mitochondria, which reduced infarct volume and cell death in the ischemia–reperfusion injured brains. FeTMPyP, a peroxynitrite decomposition catalyst, and Mdivi-1, a blocker of mitophagy activation, prevented mitophagy-induced cell death in the ischemia–reperfusion injured brain.

In hippocampal neuronal cell death caused by neonatal hypoxia–ischemia, both caspase-3-dependent and caspase-3-independent cell death pathways are activated with the concomitant induction of autophagy^63,64^. Nestin-Cre-driven conditional knockout (cKO) of *Atg7* in the nervous system prevented both caspase-dependent and caspase-independent neuronal death and reduced hippocampal damage. Interestingly, neuronal death was both caspase-dependent and caspase-independent at the neonatal stage but caspase-independent with more-pronounced autophagy levels at the adult stage. However, because mice deficient in *Atg7* undergo neurodegeneration during development, whether neuronal cell death elicited by hypoxia–ischemia is truly attributable to ACD needs further study using an inducible KO adult mouse model.

#### Psychological stress

Chronic stress or prolonged glucocorticoid administration leads to loss of hippocampal neurons and a reduction in hippocampus size^65,66^. Glucocorticoid receptors are enriched in the hippocampus^67^, and adult hippocampal neurogenesis (continuous generation of new neurons in the adult hippocampus over a lifetime) is highly susceptible to psychological stress and is greatly reduced in various models of stress^68,69^. However, most studies have failed to detect signs of apoptosis; therefore, PCD of hippocampal neurons or adult hippocampal NSCs has not been considered as a mechanism of stress-induced decline in adult hippocampal neurogenesis or hippocampal damage^70,71^. However, our recent genetic study using adult NSC-specific *Atg7*-cKO mice demonstrated that chronic restraint stress (CRS) induced ACD in adult hippocampal NSCs in vivo and in vitro (Fig. 1e)^72^. As autophagy is essential for development and tissue homeostasis, deletion of key autophagy genes in the brain from an early developmental stage causes neurodegenerative symptoms and it is difficult to explore ACD in the adult mouse brain^73–75^. To overcome this obstacle and study the role of autophagy in the effects of psychological stress on adult hippocampal NSCs, a Nestin-Cre-ERT2 mouse line was crossed with *Atg7* flox mice, and *Atg7* deletion was induced in the offspring at 7 weeks of age; these NSC-specific cKO mice (Atg7-NSC cKO mice) were subjected to CRS. Histological and electron microscopic examination revealed an increase in autophagy flux but not in apoptosis, in hippocampal NSCs. Loss of NSCs and decreases in adult neurogenesis were blocked by *Atg7* deletion^72^. Furthermore, stress-triggered anxiety and depression, as well as cognitive deficits, were effectively prevented in the Atg7-NSC cKO mice. These findings indicated that ACD is undoubtedly physiologically important in mammals and that autophagy in the adult hippocampus may provide a new therapeutic avenue for the treatment of stress-induced psychological disorders.

In adult hippocampal NSCs, serum/glucocorticoid regulated kinase (SGK) family proteins are suspected to be the signaling mechanism mediating stress-induced ACD, as SGK1 was previously reported to mediate glucocorticoid effects on hippocampal neurogenesis^76,77^. The SGK family consists of three members: SGK1, SGK2, and SGK3^78^. The SGK family has a three-dimensional structure and sequence similar to those of the protein kinase B (PKB)/AKT family^78,79^. Importantly, a series of experiments using the CRISPR/CAS9 genome editing technique to knock out SGK1, 2, or 3 revealed that SGK3, but not SGK1 or 2, is a critical mediator of ACD (Fig. 2)^72^. SGK3 contains a complete Phox homology (PX) domain^78,80^, which contains a phosphoinositide-binding site. Phosphatidylinositol 3-phosphate (PtdIns3P) is the most common lipid that binds to the PX domain, and it is enriched in endosomes and vacuoles; SGK3 binds PtdIns3P and is located mostly in endosomes^79^. PtdIns3P is a product of PI3K and regulates the initiation of autophagy^81^. A point mutation in which Arg-90 is changed to Ala-90 in SGK3 prevented ACD^72^. Therefore, SGK3 is a critical regulator of stress-induced ACD and has this role by interacting with PtdIns3P in adult hippocampal NSCs. However, additional studies are required to elucidate the details of how SGK3 regulates ACD and to explore SGK3 as a potential therapeutic target for stress-induced psychological disorders.

### Molecular intersection of ACD and apoptosis

Why is ACD activated in normal cells equipped with intact apoptosis capability? This question can be answered by examining the molecular pathways that link ACD and apoptosis. Detailed studies on insulin-deficient adult hippocampal NSCs have offered a few glimpses into the complicated intersection of ACD and apoptosis (Fig. 2). Calpain 2 is a major calpain in adult hippocampal NSCs and was identified as a key rheostat of apoptosis with respect to ACD, as suppression of calpain activity promoted ACD, whereas higher calpain activity switched the cell death program from ACD to apoptosis in insulin-deprived adult hippocampal NSCs^82^. Another interesting effector in the interplay between apoptosis and ACD in adult hippocampal NSCs is valosin-containing protein (VCP), which positively regulates autophagosome maturation at the basal state. However, under conditions of high autophagy flux following insulin withdrawal, VCP regulates the autophagy initiation step^83^. Of interest, pharmacological, and genetic inactivation of VCP led to apoptosis with a concomitant increase in calpain 2 levels in insulin-deprived adult hippocampal NSCs^83^. However, the switch from ACD to apoptosis and upregulation of calpain activity by inhibition or knockdown of VCP under insulin-deprived conditions were prevented by *Atg7* knockdown, indicating that ACD is a prerequisite for the switch to apoptosis.

ATG5 and Beclin-1 were reported as substrates of calpain. Calpain cleaves ATG5 in HeLa, Jurkat, and MDA-MA-231 cells in response to several apoptotic stimuli, including etoposide, doxorubicin, and staurosporine^84^. Cleaved ATG5 then translocates from the cytosol to mitochondria, where it associates with Bcl-X1, and triggers cytochrome c release and caspase activation. Calpain-mediated cleavage of Beclin-1 following renal ischemia results in autophagy inhibition and extensive neuronal death^85^. However, we could not detect cleavage of ATG5 or Beclin-1 in insulin-deprived adult hippocampal NSCs. Therefore, understanding the molecular mechanism by which calpain regulates the switch from ACD to apoptosis in adult NSCs awaits further study.

The complex relationship between autophagy and apoptosis depends on the biological context and is not yet fully understood. Intriguingly, the two pathways share common components, such as Bcl-2 family proteins. Bcl-2 can directly bind to Beclin-1. As Beclin-1 is a core component of the VPS34 complex, which is required for phagophore formation and initiation of autophagy through the generation of PtdIns3P, the binding ability of Bcl-2 to Beclin-1 confers, in addition to its well-known antiapoptotic function, another critical cellular function to Bcl-2: an antiautophagic role. Interestingly, the interaction of Bcl-2 with Beclin-1 does not interfere with the antiapoptotic potential of Bcl-2^86^. However, this interaction can be disrupted by posttranslational modification of Bcl-2 or Beclin-1, including phosphorylation, ubiquitination, or caspase-mediated cleavage^87,88^. Nevertheless, whether the interaction of ACD with apoptosis is controlled by Bcl-2 family proteins is not yet clear. This indication will be worth more attention in the near future.

Our recent finding that caspase-9 is activated in an APAF-1-independent manner in insulin-deprived adult hippocampal NSCs provides another intriguing illustration of the interaction of autophagy and apoptosis^89^. Caspase-9 promotes ACD but not apoptosis following insulin withdrawal in adult NSCs^89^. Elucidation of the molecular mechanism by which autophagy directs caspase-9 into ACD rather than apoptosis will greatly advance our understanding of the interconnection between apoptosis and ACD.

## Conclusion and unresolved questions

Studies on the cell death mechanism in adult hippocampal NSCs following insulin withdrawal or psychological stress have greatly contributed to the elucidation of ACD at the molecular level. Adult hippocampal NSCs have intact machinery for apoptosis and necroptosis subroutines, as indicated by staurosporine or H_2_O_2_ treatment inducing apoptosis or necroptosis in these cells, and this machinery can be inhibited by appropriate pharmacological inhibitors^72^. Therefore, the immediately forthcoming question is how ACD rather than apoptosis/necroptosis is predominantly triggered to promote cell death. Another conundrum is the nature of the signaling mechanisms that dictate the contradictory roles of autophagy in cell death and cell survival. As a compromise, it has been assumed that basal, low-level autophagy is cytoprotective, whereas the sustained excessive level of autophagy flux causes cell death. However, this assumption has not yet been tested experimentally. As most techniques to measure autophagy flux are qualitative, quantitative comparisons of autophagy flux between different conditions, even in the same cell type, as well as between different cell types, is technically very challenging. Therefore, the molecular mechanisms of ACD are far from being understood. Nevertheless, we have recently witnessed an increasing recognition of the critical roles of ACD in mammalian pathophysiology, including tumor suppression and mental disorders associated with psychological stress. Elucidation of this uniquely programmed mechanism of cell death holds great potential for applications of autophagy in human health and the treatment of diseases.
